# Honey bee (*Apis mellifera ligustica*) acetylcholinesterase enzyme activity and aversive conditioning following aluminum trichloride exposure

**DOI:** 10.1186/s40850-021-00103-8

**Published:** 2022-01-12

**Authors:** A. M. Chicas-Mosier, T. E. Black, K. P. Hester, L. P. Belzunces, C. I. Abramson

**Affiliations:** 1 Department of Entomology and Plant Pathology, Auburn University, Auburn, AL USA; 2 Department of Psychological Sciences, Weber State University, UT Ogden, USA; 3 Department of Physiological Sciences, Oklahoma State University, Stillwater, OK USA; 4 INRAE- Abeilles et Environnement, Avignon, France; 5 Department of Integrative Biology, Oklahoma State University, OK Stillwater, USA; 6 Department of Psychology, Oklahoma State University, 116 Psychology Building, Stillwater, OK USA 74078

**Keywords:** Aversive conditioning, Honey bee, Aluminum trichloride, Acetylcholinesterase

## Abstract

**Background:**

Aluminum is the third most prevalent element in the earth’s crust. In most conditions, it is tightly bound to form inaccessible compounds, however in low soil pH, the ionized form of aluminum can be taken up by plant roots and distributed throughout the plant tissue. Following this uptake, nectar and pollen concentrations in low soil pH regions can reach nearly 300 mg/kg. Inhibition of acetylcholinesterase (AChE) has been demonstrated following aluminum exposure in mammal and aquatic invertebrate species. In honey bees, behaviors consistent with AChE inhibition have been previously recorded; however, the physiological mechanism has not been tested, nor has aversive conditioning.

**Results:**

This article presents results of ingested aqueous aluminum chloride exposure on AChE as well as acute exposure effects on aversive conditioning in an *Apis mellifera ligustica* hive. Contrary to previous findings, AChE activity significantly increased as compared to controls following exposure to 300 mg/L Al^3+^. In aversive conditioning studies, using an automated shuttlebox, there were time and dose-dependent effects on learning and reduced movement following 75 and 300 mg/L exposures.

**Conclusions:**

These findings, in comparison to previous studies, suggest that aluminum toxicity in honey bees may depend on exposure period, subspecies, and study metrics. Further studies are encouraged at the moderate-high exposure concentrations as there may be multiple variables that affect toxicity which should be teased apart further.

**Supplementary Information:**

The online version contains supplementary material available at 10.1186/s40850-021-00103-8.

## Background

Aluminum is the third most prevalent element in the earth’s crust [36]. Typically, it is tightly bound in compounds such as aluminum hydroxide; however modern anthropological changes have made the element increasingly bioavailable through ionization [40]. Decreases in soil pH following bauxite mining, or acidification events such as rain or fertilizer application, can increase aluminum bioavailability, especially to plant roots [5, 29, 51]. In North America, concentrations in plant tissues have been found between 0.5 mg/kg and 670 mg/kg [2, 3], with pollen concentrations up to 268 mg/kg in contaminated regions of Brazil [32]. In comparison, though dissolved, the aluminum limit for bottled water is 0.2 mg/L in the United States [2, 3]. Given the concentrations of aluminum observed in plant products, organisms with direct interaction or ingestion of the contaminated produce, such as pollinators, may be at risk.

Research has shown aluminum increases mortality and has dose-dependent effects on motility in *Apis mellifera mellifera*; additional literature suggests impacts on flower color choice bias in *A.m. mellifera*, *A.m. carnica/caucasica*, and *A.m. mellifera/scutellata* [13, 14]. Evidence in bumble bees (*Bombus terrestris audax*) has shown bioaccumulation in larvae without taste aversion to the metal [20, 31]. Although behavioral data exists, there has yet to be confirmation that the same mechanism of neuro-disruption observed in mammals, acetylcholinesterase activity inhibition, also occurs in terrestrial insects in response to aluminum exposure nor is there definitive aversive conditioning data [13, 14, 28, 30, 48].

The cholinergic system relies on the enzyme acetylcholinesterase (AChE), to degrade acetylcholine through hydrolysis for successful synaptic reuptake. This system is well studied in mammals, invertebrates, birds, and fish [34]. Degradation of AChE can result in over-binding of acetylcholine and subsequently overstimulation of postsynaptic neurons. Overstimulation can cause hyperkinesia, memory deficits, and an overactive autonomic nervous system [15, 26, 45, 46]. Effects of aluminum on AChE warrants additional study using arthropods because insect models have been used to ethically and experimentally study neurological deficits with human health implications [1, 41], and insects are experiencing global decline with particular concern for pollinator species [22, 24]. In addition, determining if AChE disruption is occurring in insects would provide a mechanistic explanation of previously recorded behavioral data [13, 14].

Documented population decline in nectarivorous insects, such as bees, is attributed to three primary factors: habitat fragmentation, pesticide application, and pathogens [22, 23]. However, the research focus on pesticide application may be too narrow, as it does not cover anthropogenic effects that cause increased metal exposure such as mining and acidification [39]. Limited evidence suggests a reduced response to aversive stimuli in aluminum trichloride exposed honey bees; specifically, returning to a potentially hazardous environment [13]. Considering the concentrations of aluminum in plant matter, the risks of exposure, subsequent negative health effects, and limited survival, understanding aluminum exposure is valuable [5, 39].

The United States and Europe have multi-billion dollar economies fueled by pollinators and the services they provide [21, 25]. The economic importance of honey bee populations, in addition to human dependence on pollination services for food security, makes understanding their decline extremely important. In areas where soil is highly acidified through anthropogenic impacts, the available aluminum concentrations in plant tissues are expected to be high enough to cause chronic hive-wide effects in honey bees [10, 13, 14]. Compared to the concentrations found by the U.S. ATSDR in plant materials, and pollen concentrations previously found in Brazil (up to 670 mg/kg), the concentrations that appear to cause behavioral deficits in honey bees are relatively low (10.4 mg/L) ([2, 3, 13, 14, 32];). Although these concentrations are not directly comparable because of ingestion route, aqueous versus food sources, consumption through water is an established method and is considered a conservative approach [14].

This article seeks to understand how aqueous aluminum trichloride affects honey bee physiology (AChE enzyme activity) and subsequent behavior (aversive conditioning). Hypothetical outcomes, based on previous literature and limited free-flight data, are decreased avoidance of aversive stimuli and AChE analysis may reveal hormetic or decreasing enzyme activity with increasing exposure [13, 14].

## Results

### Acetylcholinesterase enzyme activity

**Fig. 1:**
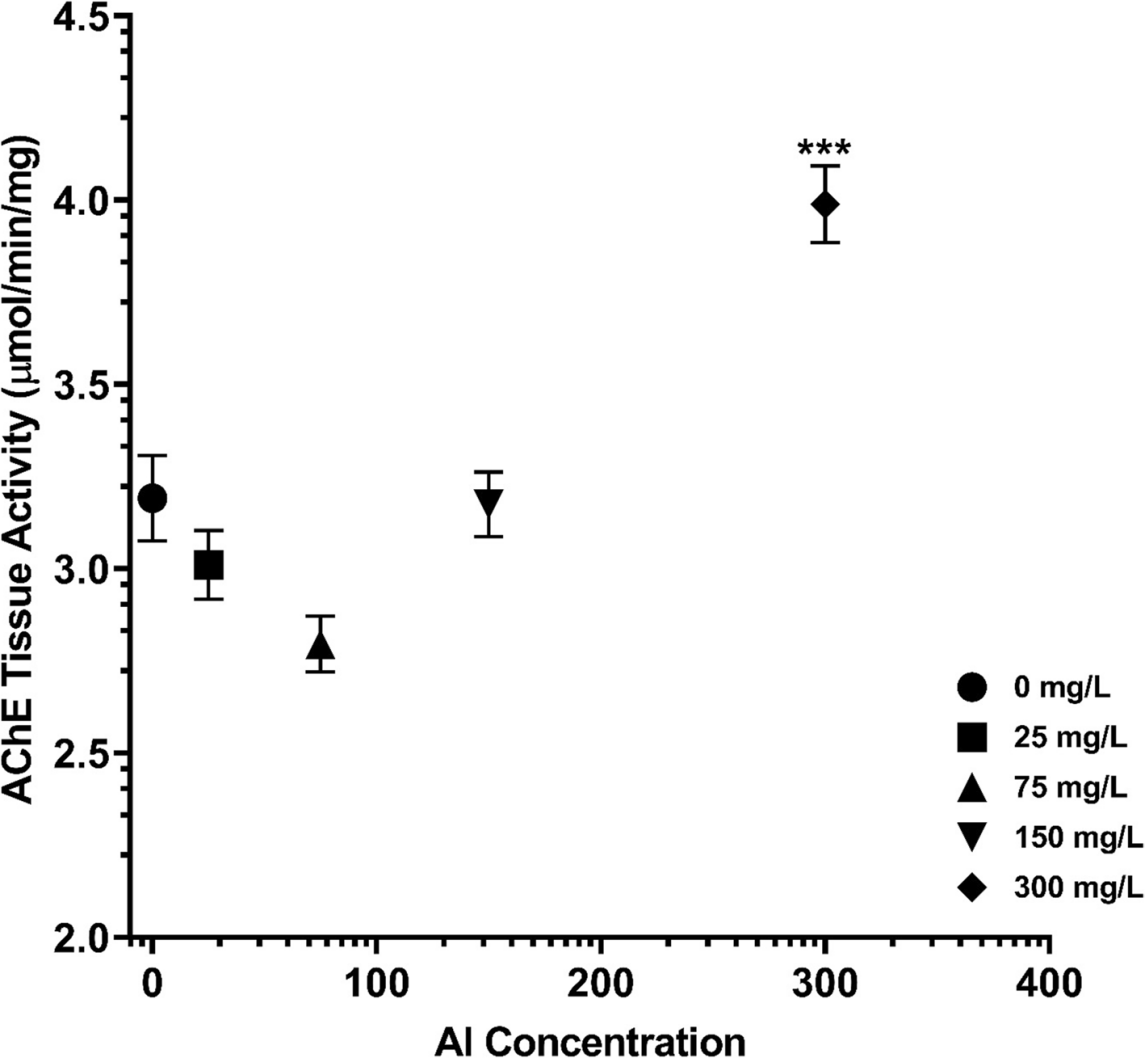
AChE tissue activity by aluminum concentration on log-scale (±SE, ***p < 0.0001 from 0 mg/L)

Analyses of variance were run within subspecies followed by Tukey *post-hoc* tests to compare each concentration mean (ANOVA: F(4,203) =20.34, *p* < 0.0001, Table 1). The highest exposure concentration, 300 mg/L, increased AChE enzyme activity significantly compared to all other exposure concentrations, including controls (Fig. 1). The other significant comparisons were a decrease in enzyme activity between 0 mg/L and 75 mg/L and an increase between 75 mg/L and 150 mg/L exposure concentrations (Table 1).

**Table 1:** Tukey-Kramer HSD post-hoc significance table (p < 0.05) for AChE enzyme activity

Aluminum Concentration		0 mg/L	25 mg/L	75 mg/L	150 mg/L	300 mg/L
Comparative Aluminum Concentration	25 mg/L	NS				
	75 mg/L	*p* = 0.0343	NS			
	150 mg/L	NS	NS	*p* = 0.0406		
	300 mg/L	p < 0.0001	p < 0.0001	p < 0.0001	p < 0.0001	

NS are non-significant results (*p* > 0.05)

### Gel electrophoresis

Gels were analyzed for bands to detect soluble versus insoluble AChE. There was negligible detection of the soluble form of AChE and no detection variation within exposure concentrations. This implies that the honey bee AChE activity results are driven almost entirely by the membrane bound form of the enzyme. Images of the gels are included as supplemental information.

### Aversive conditioning

#### Centerline crossings

**Fig. 2:**
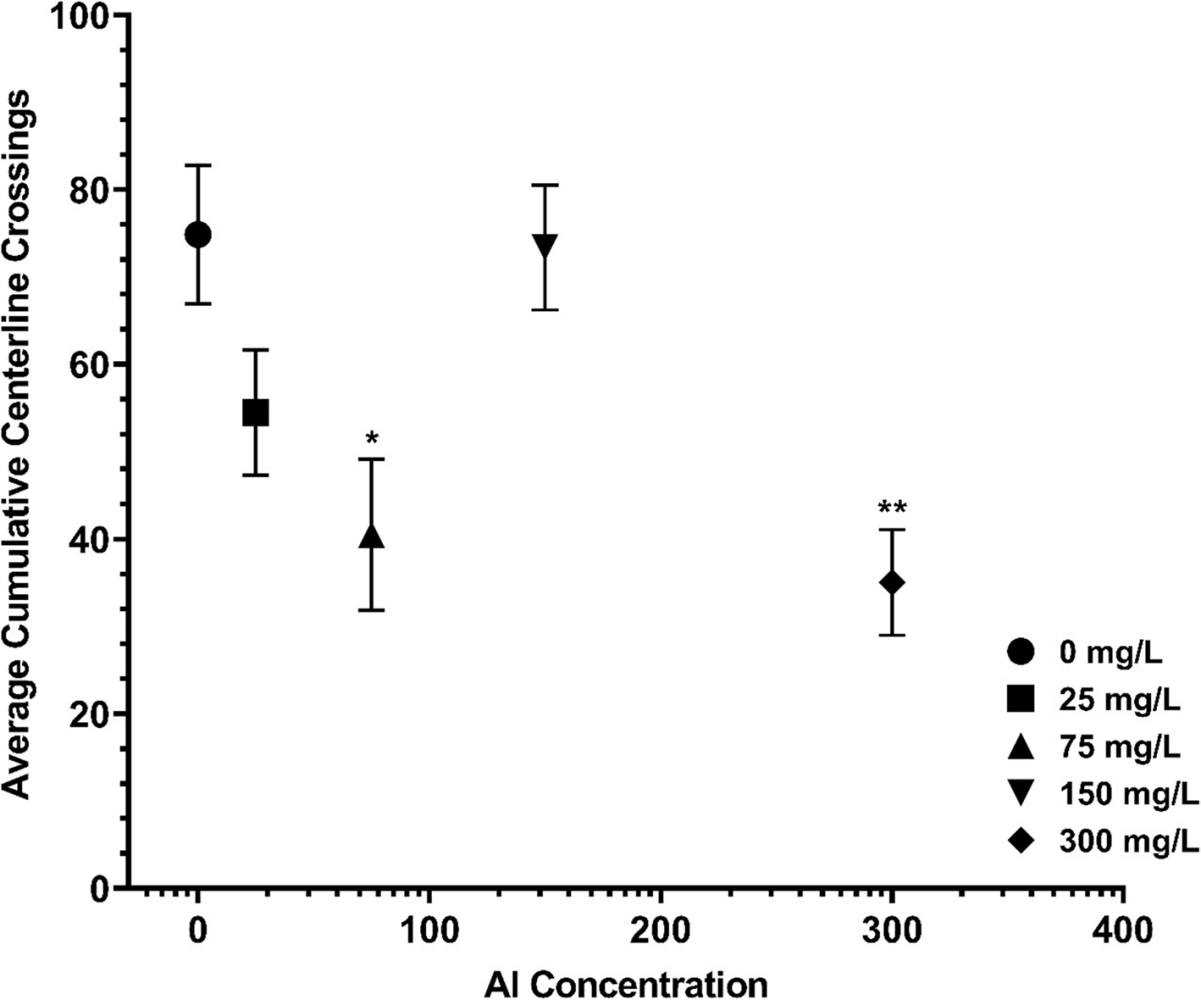
Average number of centerline crossings, an indicator of motility during the aversive conditioning experiment, by concentration (±SE, *p < 0.05, **p < 0.01 from 0 mg/L)

Analysis of variance (ANOVA) showed significant effects on motility, observed as the cumulative number of centerline crossings, following exposure to Al^3+^ as compared to no-treatment controls (F(4,58) = 5.34, *p* = 0.001, Fig. 2). Dunnett’s test for multiple comparisons revealed that there was significantly reduced activity following 75 mg/L (*p* = 0.012) and 300 mg/L exposure concentrations (*p* = 0.002).

#### Deviation from baselines by role

**Fig. 3:**
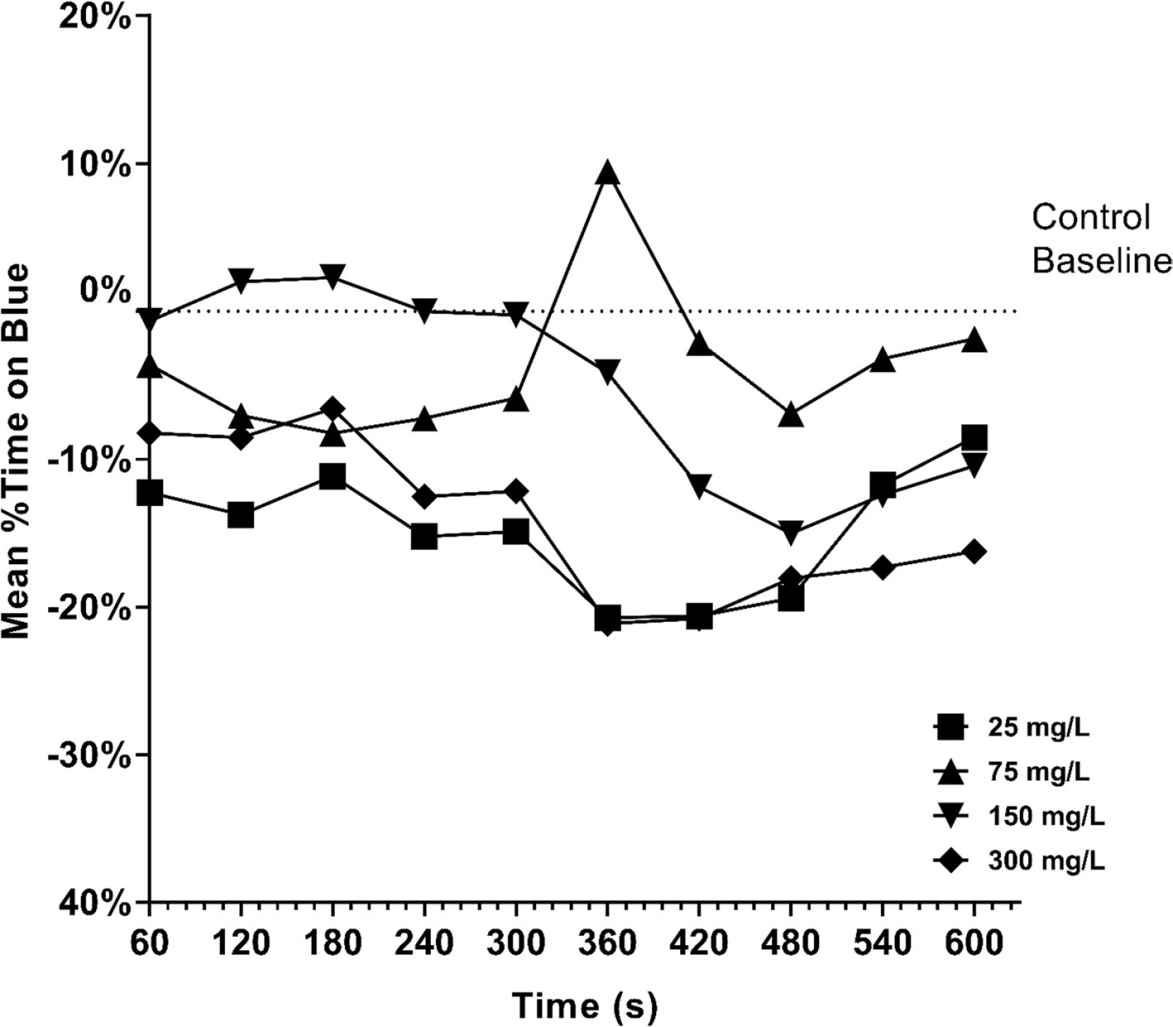
Mean percent time spent on the blue half of the shuttlebox during baseline trials. The dotted line is the 0-baseline values as determined in Eq. 1

Baseline role bees are only exposed to color stimuli. One sample t-tests from a hypothetical mean of 50% blue visitation showed significant yellow bias regardless of the exposure concentration (5.221 < t < 23.41, df = 9, *p* ≤ 0.0005). One-sample t-tests of Al^3+^ exposed baseline honey bees compared to 0-baseline controls showed significant deviation after 25, 150, and 300 mg/L but not 75 mg/L (Fig. 3, Table 2).

**Table 2:** Significance table for shuttlebox by honey bee role. Comparisons were made to 0-baseline bees

Role	Side Shock	Al^3+^ Conc. (mg/L)	P	T
Baseline	None	0^a^		
		25	< 0.0001	11.13
		75	NS	
		150	0.040	2.4
		300	< 0.0001	8.46
Learner	Blue	0	0.001	4.63
		25	0.016	2.98
		75	NS	
		150	0.041	2.38
		300	0.026	2.66
	Yellow	0	0.020	2.789
		25	NS	
		75	0.017	2.91
		150	0.001	5.34
		300	NS	
Matched	Blue^b^	0	NS	
		25	0.000	5.79
		75	0.008	3.42
		150	0.001	5.2
		300	< 0.0001	23.79
	Yellow^b^	0	< 0.0001	6.96
		25	< 0.0001	8.77
		75	0.040	2.41
		150	< 0.0001	7.32
		300	< 0.0001	8.9

NS are non-significant comparisons (p > 0.05) and all df = 9. In all significant comparisons mean time on blue decreased

^a^0-baseline bees are the comparative group so no comparisons were run

^b^Matched bees could be shocked on either color; as the randomized control, shock was dependent on the paired learner bee’s location

**Fig. 4:**
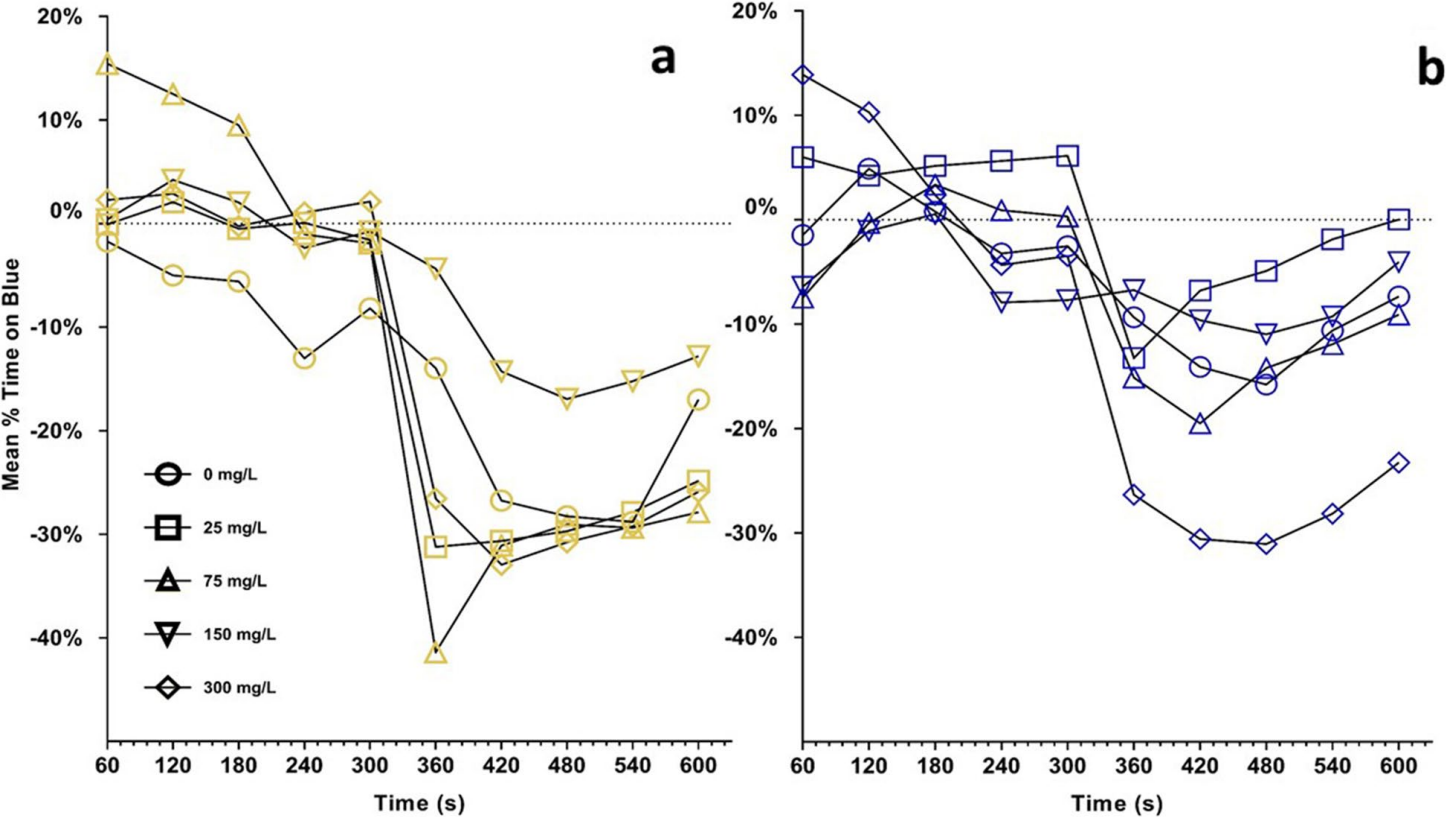
Results of the learner trial bees for the two colors offered. The dotted line for each figure is the 0-baseline values as determined in Eq. 1. The trend change that appears at 300 s was not the result of any experimental modification and time effects were non-significant. **a**: Mean percent time on the blue half of the shuttlebox when yellow was safe (non-shock). **b**: Mean percent time on the blue half of the shuttlebox when blue was safe (non-shock)

One-sample t-tests from 0-baseline controls in learner bees aggregated across time showed significant deviation when the blue side contained shock after 0, 25, 150 and 300 mg/L exposure concentrations but not 75 mg/L (Fig. 4a). When the yellow side held shock, significant deviation occurred from 0-baselines following 0, 75, 150 but not 25 or 300 mg/L exposure concentrations (Table 2, Fig. 4b).

**Fig. 5:**
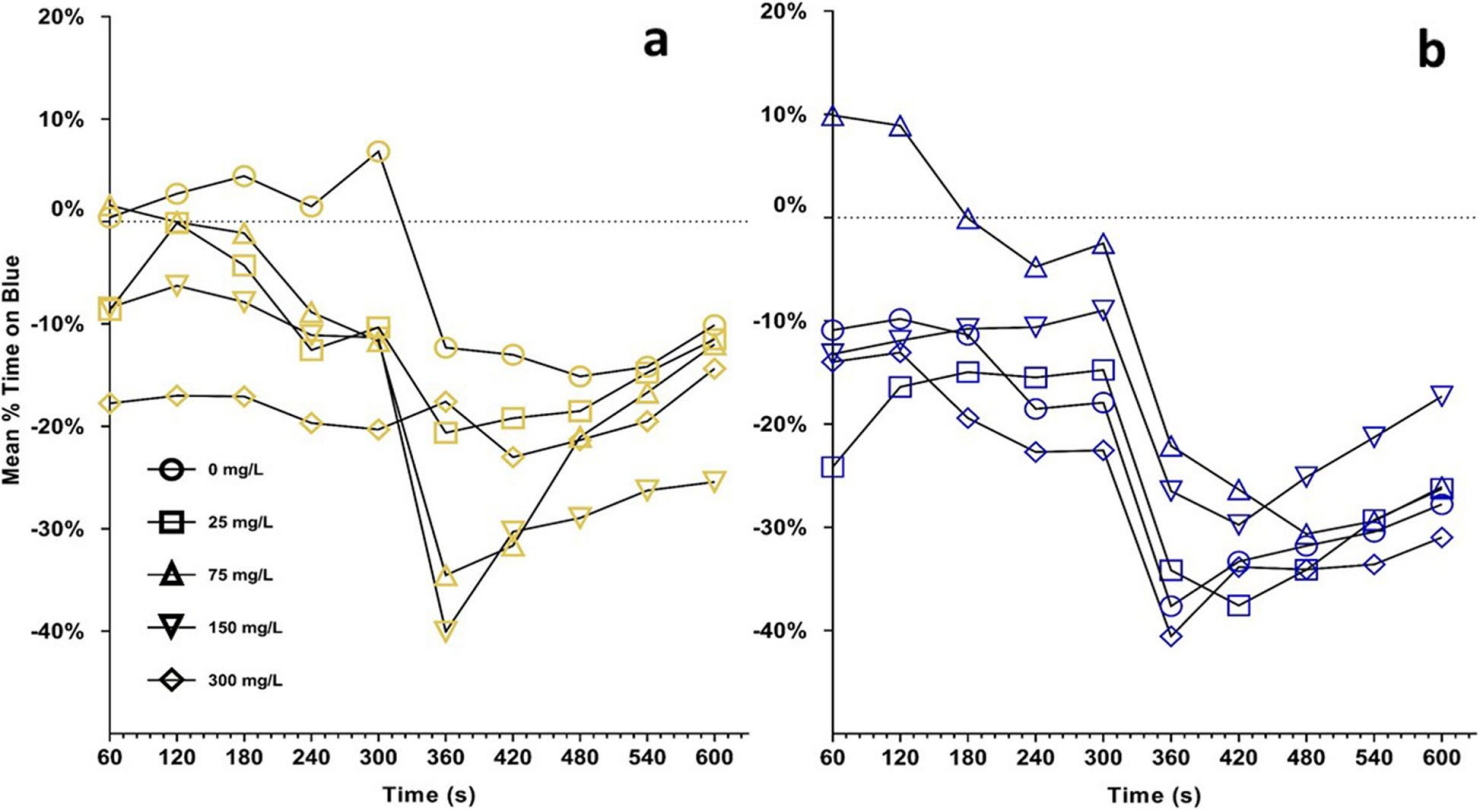
Results of the matched trial bees for the two colors offered. The dotted line for each figure is the 0-baseline values as determined in Eq. 1. The trend change that appears at 300 s was not the result of any experimental modification and time effects were non-significant. **a**: Mean percent time on the blue half of the shuttlebox when yellow was safe (non-shock). **b**: Mean percent time on the blue half of the shuttlebox when blue was safe (non-shock)

For matched bees, when the blue side held shock for the paired learner bee, there was significant deviation from 0-baselines following all exposures except controls (Fig. 5a). When yellow was shocked, significant deviation occurred following all exposure concentrations (Table 2, Fig. 5b). Overall, regardless of honey bee role, significant results indicated a reduced percent time on the blue color stimulus, indicating a stronger adherence to preexisting color bias following exposure.

## Discussion

### Physiology

The physiological and behavioral data presented here demonstrate that exposure duration may impact the severity of response to aluminum exposure. Considering the physiological data, *Apis mellifera ligustica* honey bees showed an unexpected trend in AChE enzyme activity. Most exposure concentrations did not change enzyme activity significantly following exposure to aluminum, however a spike in enzyme activity was seen at the highest exposure concentration. This is in contrast to other studies of aluminum exposure in the literature which have found inhibition of AChE in other species and reduced motility in honey bees [14, 30, 35, 47].

Although the 300 mg/L exposure concentration did not match previous observations, the overall curvature of the enzyme activity does match that of previous motility data, but to a lesser extent [14]. The comparative increase in enzymatic activity between 75 mg/L and 150 mg/L observed in the present article is the inverse of previously reported behavioral data in *Apis mellifera mellifera* in which motility over the course of 14 days decreased between 75 and 134 mg/L exposure concentrations [14]. As AChE is the degradation enzyme of acetylcholine, a neurotransmitter which is integral to movement in honey bees, it is expected that increased activity and AChE enzyme activity would have such as inverse relationship.

In Chicas-Mosier et al. [14], motility following exposure to ≥134 mg/L was not significant compared to control values (*p* > 0.05). Based on those results, the present article hypothesized that control, 150, and 300 mg/L AChE values would either be non-significant or there would be an incremental increase in AChE enzyme activity with increasing exposure concentration (≥134 mg/L). However, the only significant increase, as compared to controls, in enzyme activity followed exposure to 300 mg/L Al. The dramatic increase in enzyme activity following 300 mg/L (*p* < 0.001) does not match the original hypotheses or the established literature. For this reason, further comparisons with multiple hives and between honey bee subspecies will need to be conducted to unravel the intricacies of intoxication at concentrations ≥134 mg/L.

In terms of impacts of aluminum from the present physiological analysis, exposure above 134 mg/L could reduce foraging success and therefore colony survival through lethargy. Such high AChE activity as compared to control honey bees, could drastically reduce movement in and outside of the hive; this is further demonstrated by the reduced centerline crossings in the shuttlebox. Given the food sharing ecology of honey bees, increased aluminum in floral products could cause neurodegeneration across the castes, resulting in hive death [27]. However, this is limited by the genetic similarities within the single hive tested here. Physiological responses may change with genotypic variation between hives and reduce the effect. Further investigation is needed to determine if this trend is consistent within subspecies.

### Behavior

In the aversive conditioning apparatus, the data matched prior honey bee experiments with lower motility than controls following short-term aluminum exposure of 300 mg/L with a decrease from controls also occurring following 75 mg/L. As the motility data demonstrated here, and has been previously recorded with chronic exposure in Chicas-Mosier et al. [14], there are similarities but some exposure concentrations caused distinct variation. The three metrics; monitor system (*A.m. mellifera*) [14], shuttlebox apparatus, and AChE enzyme activity (*A.m. ligustica*), used different exposure durations, 2 weeks, 30 min, and 48 h, respectively, with similar but diverging results. This implies that the disparate outcomes may be due in part to exposure duration as well as subspecies and hive-level variation. Exposure duration may also partially explain conflicting mammalian literature that has attempted to understand AChE following aluminum exposure. Some previous explanations for inhibition and activation effects have been brain region specificity and in vitro versus in vivo comparisons; however, the present study suggests that dosing procedures may also play a role [33, 35, 47, 49, 50].

Color-bias adherence has also been influenced by aluminum exposure, with subspecies playing a role. In Chicas-Mosier et al. [14] the gentle Africanized honey bee hybrid (*Apis mellifera mellifera/scutellata*) of Puerto Rico [37], appeared to abandon their yellow preference following exposure to 40 mg/L aluminum, however *Apis mellifera mellifera* did not show bias change at similar time scales (15–30 min). In the present study, Al^3+^ exposed baseline bees appeared to show stronger color bias as compared to controls, with 25 and 300 mg/L exposed bees spending ~ 10–20% more time in yellow than controls. For learner and matched bees, significant comparisons (Table 2) also showed decreased time spent in the blue side of the apparatus. This suggests that the *A.m. ligustica* used in this study had a stronger adherence to their preexisting color bias following exposure; this may imply a reduced ability to learn and adapt to changing floral environments which could result in lower foraging success. However, this result could also be a product of within-hive or regional variation.

### Future directions and sources of variation

Comparative analyses of a single metric with multiple hives and subspecies is needed. In the present study, a single hive was used to standardize prior toxicant exposure. As foraging honey bees provide the food resources for the entire hive, the exposure to various toxicants should be similar. This tactic has been used in previous literature studying aluminum exposure in honey bees to limit unaccounted for toxicant exposure that would be incurred by comparing multiple hives [13, 14]. This provides a look at the hive-level outcome of aluminum exposure; however, future analyses should expand the sample size with multiple hive and subspecies in a large international comparative study. This study would need to control for seasonal variation in colony demographics and use a single standardized protocol in each location to make broader conclusions regarding subspecies, colony, and individual susceptibility to aluminum.

The cholinesterase data seen in the present study may be a product of the short exposure period or may be reflective of the tolerance mechanism of this subspecies or colony. Further analysis with *A.m. ligustica* subspecies is needed to determine if the significant increase in AChE activity holds across colonies and exposure periods. The present data, suggests lower susceptibility to small changes in soil pH and aluminum exposure in the hive studied. However, at high exposure concentrations, such a dramatic increase in AChE activity may limit organismal survival through lethargy. Previous and present behavioral data suggest that aluminum exposure significantly affects movement in bees in as little as 20 min [13, 19]. With this sort of rapid intoxication, aluminum exposure warrants further study at the hive, subspecies. and species levels.

Bees in the AChE experiment were dosed for 48 h in their water supply before termination and freezing. This may not fully illuminate the effects of chronic aluminum exposure in honey bees. Based on previous literature using the same dosing procedure and longer exposure times, we would anticipate eventual lower motility and higher mortality from induced paralysis [14]. Although adaptation to higher bioavailable aluminum concentrations is possible, it would require genetic variation that is limited among European honey bees [18]. It is expected that during paralysis events, the AChE activity would be much lower than what is demonstrated here. This study did not look into longer exposure times, as bees were required to be alive when anesthetized to generate the most reliable data for the AChE analysis. Although longer dosing experiments are possible, previous experiments have shown a significant decrease in longevity in captive experiments following relatively low aluminum exposure concentrations (~ 10 mg/L), reducing the overall feasibility of such a study [14].

Dosing protocols are also a potential source of variation when comparing across experiments. In the above AChE experiment, the dosing metric was deliberate to compare across similar studies that have used honey bee subspecies in different global regions [14]. This method has shown variation between honey bee subspecies following aluminum exposure and maintaining this technique improves comparability between studies with increased data quality as compared to mass-dosing through bee cages. In the aversive conditioning experiment, sponges were used to dose individual honey bees similarly to filter papers in the monitors system. Considering the results between exposure concentrations, which match previous studies, and observation of the honey bees actively feeding on these sponges, this exposure technique was deemed effective in dosing the subjects with the aluminum trichloride solution.

## Conclusions

The present study, in tandem with other studies, suggest that motility, AChE enzyme activity, and color bias adherence are affected by aluminum exposure; however, the extent of intoxication may be dependent on exposure duration, subspecies, and individual or hive-level variation. The use of honey bees to estimate the effects of toxicant exposure on other insect species may be inadequate given the variable toxicity effects between the present data and previous publications [13, 14]. These findings imply that intoxication from indirect pollution, especially of metals such as aluminum, is worthy of further study. Further analysis of aluminum exposure is needed to determine how populations of honey bees may be impacted by aluminum exposure.

## Methods

### Subjects

*Apis mellifera ligustica* near Avignon, France were used for both studies. Hives were maintained and artificially bred for subspecies lineage by beekeepers at Institut National de la Recherche Agronomique (INRA). All honey bees were collected from the same queen-right 10-frame Langstroth hive and were assumed to be foragers (> 21 days old) [38]. Pollen and nectar foraging bees were caught by adhering cheesecloth to the entrance of the hive. The returning pollen and nectar foragers were caught in the cheesecloth. After approximately 20 bees were enmeshed in the cloth, it was removed from the hive and bees were transferred to an INRA hoarding cage [44] containing bee candy (1:2 honey sucrose paste, Chicas-Mosier [14]) until they could be moved to individual 15 mL falcon tubes. This process was completed 1–2 times daily to limit honey bee health decline or death.

### Acetylcholinesterase assay

#### Trikinetics monitor dosing procedure

Thirty-two 15 mL falcon tubes, each containing one honey bee, were loaded into the monitor apparatus. Details for this apparatus and honey bee loading are described in Chicas-Mosier et al. [14]. The Trikinetics monitors have an automated data collection feature which was not used; rather, the monitors were used as a method of exposure that matched prior aluminum studies in honey bees. Each 15 mL falcon tube lid contained a small amount of bee candy that was covered with a 1 × 1 cm layer of cheesecloth to prevent bees from becoming adhered to the food [14]. The monitor system passively delivers water to each falcon tube through a filter paper connected to a chlorinated polyvinyl chloride (CPVC) pipe containing either deionized water (DI) or the experimental solution: DI with dissolved aluminum trichloride.

Aluminum trichloride was delivered at 5x the concentration of aluminum needed to account for the weight of the chloride. A previous search of the literature did not implicate chloride as a potential behavioral modifier in this form and this dosing technique is standard in aluminum exposure studies [4, 12–14, 28]. Aluminum concentrations were 25, 75, 150, and 300 mg/L. Monitors were kept in complete darkness for 48 h at 35^o^ C to mimic a hive-like environment. After 48 h, the bees were removed from the incubator. Living bees were anesthetized in a freezer before being consolidated for storage (−20^o^ C for < 4 weeks).

#### Tissue preparation

Frozen honey bee heads were removed with a scalpel and placed in triplicate into a pre-weighed 2 mL Eppendorf tube (n_25_ = 11, n_75_ = 10, n_150_ = 11, n_300_ = 10, n_control_ = 10). Tubes were then weighed a second time to determine head weight and the amount of extraction solution needed (10% w/v). Bee heads were homogenized in an extraction solution of Triton X-100, LS-Phosphate pH 7.4 and a trypsin inhibitor solution of pepstatin, leupeptin, aprotinin, soybean trypsin inhibitor and antipain. Homogenization was conducted using steel beads in a Qiagen Tissue Lyser II. Tissues were lysed in two rounds, each round consisted of five periods of 10 s at 30 Hz. Periods were separated by 30 s rest intervals. Between the two rounds, Eppendorf tubes were chilled at 4 °C for 10 min to counteract vibrational heating.

Following homogenization, all samples were centrifuged at 15000 g for 20 min at 4^o^ C. After centrifugation, the supernatants were collected and placed in pre-labelled 1.5 mL Eppendorf tubes and used immediately for spectrophotometry. Five replicates of 5 μL of each supernatant were pipetted into a flat-bottom 96-well plate. Using a multi-channel pipette, each well was then filled with 195 μL of coloration solution consisting of LS-phosphate, pH 7 with acetylthiocholine and 5,5′- Dithiobis (2-nitrobenzoic acid). Immediately after the coloration solution was added, each plate was run in a spectrophotometer set at 412 nm for 5 min (7 data points per well). This is the standard technique for honey bee head acetylcholinesterase analysis and has been used with other toxicants previously [6–9].

#### Gel electrophoresis

Gels were made using 2X glycine-Tris pH 8.9, 30% acrylamide solution, 10% Triton-X, Tetramethylethylenediamine, and ammonium persulfate 10% solution for slow polymerization. Gels were 0.75 mm thick and in a sample buffer of glycerol, 2x glycine- Tris pH 8.9, saturated methyl red and 10% Triton-X. Sample buffer was pipetted into the gels with head extract (same protocol as was used for the AChE assay but with 2 bee heads per concentration) in a 1:1 ratio. Electrophoresis was run at 1 V/cm for 10 min then 10 V/cm for 5 h to maximize separation between the soluble and membrane bound AChE. Gel coloration included Solution A: maleate buffer 0.2 M pH 6.0, tribasic sodium citrate 0.1 M, copper sulphate 0.030 M, and acetylthiocholine added just below coloration and Solution B: 0.0049 M potassium hexacyanoferrate (III). Solution A and Solution B were mixed just before washing the gels at a 9:1 ratio.

#### Statistical analysis

Statistical analysis was completed in SAS JMP 14 (Cary, NC) software. Analyses of variances (ANOVA) and Tukey-Kramer HSD for all pairs were used to compare concentrations.

### Aversive conditioning apparatus

The aversive conditioning assay (shuttlebox) is described in Dinges et al. [17] and Black et al. [11]. The shuttlebox consists of two compartments, a shock grid, and an operators box containing a programmed Propeller controller [42, 43]. Each runway contains two sets of infrared light-emitting diodes (LEDs) and detectors at the halfway point; movement past these beams is automatically recorded by the Propeller controller. Side-choice is paired with color stimuli using blue and yellow paint swatches positioned underneath the shock grid. Honey bees often have a bias toward blue or yellow, so selecting these colors provides additional information on learning in the presence or absence of preference [11, 14]. The shock grid was attached to a power supply set to deliver 6 V at 0.5 A to the entire shock grid (except during baseline trials) as determined by the Propeller controller.

#### Aversive conditioning dosing procedure

Following honey bee collection (see *Subjects*), bees were removed from INRA hoarding cages [44] and placed individually into 15 mL falcon tubes. Each falcon tube contained a ~ 1 × 1 cm sponge soaked in 250 μL of 1 M sucrose with or without aluminum trichloride treatment. The same concentrations used for the AChE experiment (0, 25, 75, 150 and 300 mg/L, Table 3) were used. Bees were allowed to feed from the sponge ad libitum for 30 min before testing. Bees that did not feed from the sponge during the 30 min exposure phase were not included in the study.

**Table 3:** Description of aversive conditioning roles and sample sizes for each concentration

	Learning Expected	Shock Color	Purpose	Exposure Concentration (mg/L)				
				0	25	75	150	300
				Sample Size (number of bees)				
Baseline	No	None	Determine color bias and deviation with AL exposure	13	12	11	10	12
Learner	Yes	Blue or Yellow	Establish whether learning or aversion are impacted by AL exposure	15	12	10	13	12
Matched	No	Blue and Yellow	Randomized control to account for exhaustion effects	13	10	11	13	11

#### Aversive conditioning task

To start the experiment, a treated bee (see *Aversive Conditioning Dosing Procedure*) was placed in each shuttlebox compartment and randomly assigned to a role: learner, matched, or baseline (Table 3). Baseline bees were paired with a second baseline bee, whereas the experimental conditions followed a matched-subjects design (learner and matched, often referred to as master-yoked pairs). Following role assignment and placement into the shuttlebox, bees were allowed a recovery period (no shock stimuli or data recording) of 5 min to acclimate to the new environment. After the recovery period, and both bees had crossed the centerline, the controller began automatically collecting data for two consecutive, identical, 5 min trials. Honey bees designated as baseline served as behavioral controls, the only stimuli provided were the color swatches. Baseline bees provided data on color bias and overall changes with increasing Al^3+^ concentration. Baseline bees that were not given aluminum trichloride solution (0-baseline) served dual-control purposes and were used as the primary comparison group. During the baseline trials, the power supply was disconnected from the shock grid to ensure no additional cues were provided.

Bees designated as learners were in a paired color and shock cue environment. Safe and shock colors were counterbalanced to account for preexisting color biases. When learner bees were on the side designated as safe, the shock grid issued no shock; however, when on the other color, shock was applied to the entire grid, including to the matched bee’s compartment. Learner bees acted as the learning experimental treatment because they were the only role provided with paired stimuli (shock+ color). Matched bees served as a randomized control, this group was shocked when the paired learner bee was on the unsafe side of the apparatus. Matched bees were not expected to learn as they could receive shock regardless of the color environment. This system has been previously used to study toxicant effects on aversive learning [16].

#### Statistical analysis

Statistical analysis was completed in Graphpad Prism 8 (San Diego, CA). An ANOVA with *post-hoc* Dunnett’s test for multiple comparisons was used to test the number of centerline crossings as compared to 0-baseline bees. The data were aggregated by concentration across role to compare total movement regardless of learning environment.

One-sample t-tests were binned into 5 min trials following visual inspection of the graphs for each role; however, analysis showed no bin effects and data were aggregated across trials (10 min).

One-sample t-tests were used to compare to 0-baseline values for each role and concentration separately (Eq. 1). This type of analysis was used in Delkash-Roudsari [16] and estimates toxicant-induced deviation from a standardized value. This limits noise from seasonality (early summer vs. late summer) and individual variation. All comparisons regardless of role were made to this established baseline.

$$per\ minute\ deviation\ from\ baseline=\left[\frac{\left({n}_{xt}-{n}_{bt}\right)+\left({n}_{xt2}-{n}_{bt2}\right)}{2}\right]$$

Equation 1: w*here n is the per min bee average for a given x, x is role and concentration (learner or matched), b is the 0-baseline average, t is the min bin, and t2 is the corresponding min bin in the second identical trial.*

## Supplementary Information

**Additional file 1: SI Figure 1**: Electrophoresis to analyze the effect of aluminum on the proportions of soluble and membrane AChE. Upper band, membrane AChE; Lower band, soluble AChE. Control (left) and 25 mg/L (right) exposure concentration gel (5 replicates/concentration).

**Additional file 2: SI Figure 2**: Electrophoresis to analyze the effect of aluminum on the proportions of soluble and membrane AChE. Upper band, membrane AChE; Lower band, soluble AChE. Intermediate exposure concentrations (75 mg/L (left) and 150 mg/L (right)) gel.

**Additional file 3: SI Figure 3**: Electrophoresis to analyze the effect of aluminum on the proportions of soluble and membrane AChE. Upper band, membrane AChE; Lower band, soluble AChE. High exposure concentration (300 mg/L) gel.

## Data Availability

Data are available through FigShare: 10.6084/m9.figshare.14959296.

## Abbreviations

AChE: Acetylcholinesterase
*A.m.*: *Apis mellifera*
CPVC: Chlorinated Polyvinyl Chloride
DI: Deionized water
ANOVA: Analysis of Variance
INRA: Institut National de la Recherche Agronomique
LED: Light-Emitting Diode
