# Supplementary material for: Honey bee (Apis mellifera ligustica) acetylcholinesterase enzyme activity and aversive conditioning following aluminum trichloride exposure
Source: BMC Zool. 2022 Jan 12;7:5. doi: 10.1186/s40850-021-00103-8 (PMC10127314; doi:10.1186/s40850-021-00103-8)
Supplement: Supplementary file 1 — Additional file 1: SI Figure 1: Electrophoresis to analyze the effect of aluminum on the proportions of soluble and membrane AChE. Upper band, membrane AChE; Lower band, soluble AChE. Control (left) and 25 mg/L (right) exposure concentration gel (5 replicates/concentration). [file 40850_2021_103_MOESM1_ESM.docx]

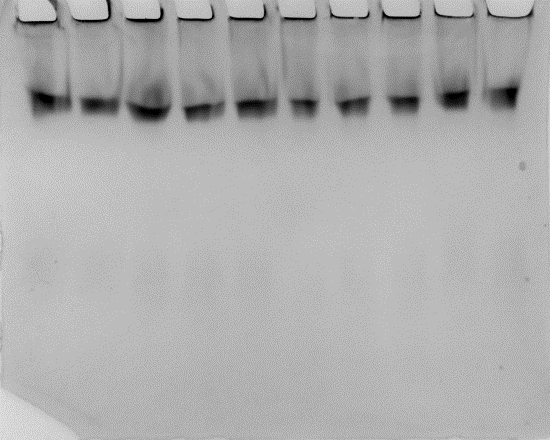


SI Figure 1: Electrophoresis to analyze the effect of aluminum on the proportions of soluble and membrane AChE. Upper band, membrane AChE; Lower band, soluble AChE. Control (left) and 25mg/L (right) exposure concentration gel (5 replicates/concentration).
