# Supplementary material for: Honey bee (Apis mellifera ligustica) acetylcholinesterase enzyme activity and aversive conditioning following aluminum trichloride exposure
Source: BMC Zool. 2022 Jan 12;7:5. doi: 10.1186/s40850-021-00103-8 (PMC10127314; doi:10.1186/s40850-021-00103-8)
Supplement: Supplementary file 2 — Additional file 2: SI Figure 2: Electrophoresis to analyze the effect of aluminum on the proportions of soluble and membrane AChE. Upper band, membrane AChE; Lower band, soluble AChE. Intermediate exposure concentrations (75 mg/L (left) and 150 mg/L (right)) gel. [file 40850_2021_103_MOESM2_ESM.docx]

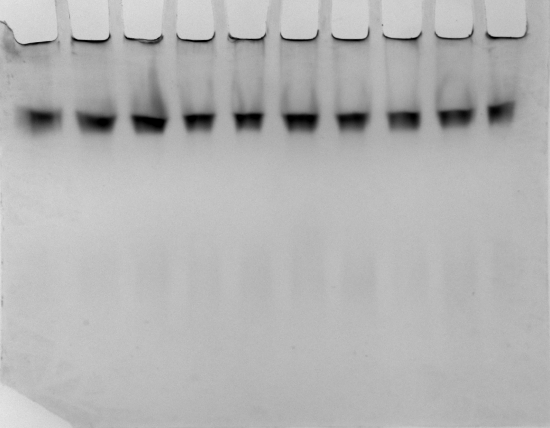


SI Figure 2: Electrophoresis to analyze the effect of aluminum on the proportions of soluble and membrane AChE. Upper band, membrane AChE; Lower band, soluble AChE. Intermediate exposure concentrations (75mg/L (left) and 150mg/L (right)) gel.
