# Supplementary material for: Honey bee (Apis mellifera ligustica) acetylcholinesterase enzyme activity and aversive conditioning following aluminum trichloride exposure
Source: BMC Zool. 2022 Jan 12;7:5. doi: 10.1186/s40850-021-00103-8 (PMC10127314; doi:10.1186/s40850-021-00103-8)
Supplement: Supplementary file 3 — Additional file 3: SI Figure 3: Electrophoresis to analyze the effect of aluminum on the proportions of soluble and membrane AChE. Upper band, membrane AChE; Lower band, soluble AChE. High exposure concentration (300 mg/L) gel. [file 40850_2021_103_MOESM3_ESM.docx]

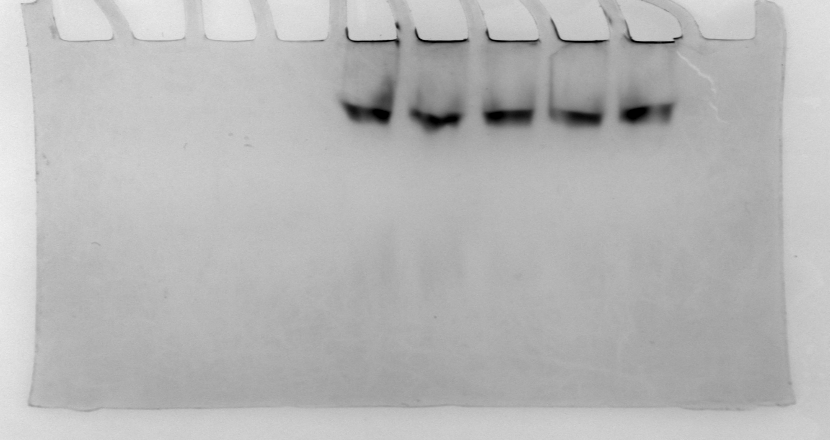


SI Figure 3: Electrophoresis to analyze the effect of aluminum on the proportions of soluble and membrane AChE. Upper band, membrane AChE; Lower band, soluble AChE. High exposure concentration (300mg/L) gel.
